# A random interacting network model for complex networks

**DOI:** 10.1038/srep18183

**Published:** 2015-12-11

**Authors:** Bedartha Goswami, Snehal M. Shekatkar, Aljoscha Rheinwalt, G. Ambika, Jürgen Kurths

**Affiliations:** 1 Potsdam Institute for Climate Impact Research, P.O. Box 60 12 03, 14412 Potsdam, Germany; 2 Indian Institute of Science Education and Research, Dr. Homi Bhabha Road, Pashan, Pune - 411008, India; 3 Institute of Earth and Environmental Science, University of Potsdam, Karl-Liebknecht-Str. 24–25, 14476 Potsdam-Golm, Germany; 4 Department of Physics, Humboldt Universität zu Berlin, Newtonstr. 15, 12489 Berlin, Germany; 5 Institute for Complex Systems and Mathematical Biology, University of Aberdeen, Aberdeen AB243UE, UK; 6 Department of Control Theory, Nizhny Novgorod State University, 603950 Nizhny Novgorod, Russia

## Abstract

We propose a RAndom Interacting Network (RAIN) model to study the interactions between a pair of complex networks. The model involves two major steps: (i) the selection of a pair of nodes, one from each network, based on intra-network node-based characteristics, and (ii) the placement of a link between selected nodes based on the similarity of their relative importance in their respective networks. Node selection is based on a selection fitness function and node linkage is based on a linkage probability defined on the linkage scores of nodes. The model allows us to relate within-network characteristics to between-network structure. We apply the model to the interaction between the USA and Schengen airline transportation networks (ATNs). Our results indicate that two mechanisms: degree-based preferential node selection and degree-assortative link placement are necessary to replicate the observed inter-network degree distributions as well as the observed inter-network assortativity. The RAIN model offers the possibility to test multiple hypotheses regarding the mechanisms underlying network interactions. It can also incorporate complex interaction topologies. Furthermore, the framework of the RAIN model is general and can be potentially adapted to various real-world complex systems.

Complex networks provide a powerful approach for investigating real-world systems. It helps us to understand phenomena observed over a wide range of disciplines such as biology, climate, environment, social sciences, technology, and economics^1^,^2^,^3^,^4^. Many real-world networks, however, are composed of subnetworks called *communities* which are more closely connected within each other than to the rest of the network^5^,^6^. In other cases, networks of one kind (e.g., a power grid) interact with networks of another kind (e.g., communications systems), leading to a necessary extension of the complex network paradigm that incorporates different types of networks with different types of interactions between them. Initial studies dealing with *interdependent networks* considered the interrelations between the Internet communication network and the power grid network^7^,^8^. There have been similar studies in other fields since then: such as that of a *network of networks* in climate^9^ where individual isobaric layers of the atmosphere were represented as a complex network with the different isobar networks connected among themselves, epidemic spreading on *interconnected networks*^10^,^11^, and that of the European air transportation *multiplex network*^12^, where each airline comprised a *layer* with the set of all airports forming a common set of nodes to all layers. For the purposes of this study, all such scenarios involving two or more networks, are examples of *interacting networks*. We use here the prefix *intra*- to denote quantities defined within a chosen subnetwork and the prefix *inter*- to denote quantities defined between subnetworks.

While modeling interacting networks most studies use a rather simple interaction structure between the subnetworks even though the topology of each subnetwork is quite complex. This is in contrast to several real-world systems where the interactions between communities are extremely relevant and complex, such as in the brain^13^,^14^. Moreover, a few studies have also indicated the influence of *intra*-network topology on the *inter*-network behavior^15^,^16^,^17^. Keeping this in mind, we put forward a much more general model of interacting networks, i.e., a RAndom Interacting Network (RAIN) model, which should offer the following features: (i) it should be able to consider any given form of interaction of each subnetwork as well as of the interactions between the subnetworks, irrespective of its complexity, (ii) it should be able to consider the dependence (if any) of inter-network interaction on the intra-network topology, (iii) it should be easily adaptable to different scenarios corresponding to different classes of real-world systems, and (iv) lastly, it should help us to understand the mechanisms that govern the formation of observed interaction structures in the systems being studied with this model. The last point is crucial as a model is useful only in as much as it extends our understanding of the systems under consideration. This is seen, e.g., in the seminal studies that revealed the small-world^18^ and scale-free^19^ behavior of single networks in real-world systems.

In this study, we first formulate the general characteristics of the RAIN model and thereafter use it to understand the interactions between the regional airline transportation networks (ATNs) from USA and the Schengen area with the idea that this not only helps to demonstrate the utility of the model framework but also sheds light on the USA-Schengen inter-network structure. ATNs have been studied considerably because of their economic and social impacts^20^,^21^,^22^, as well as their potential role in the spreading of contagious diseases^23^,^24^. Based on the single network perspective (regional as well as global), earlier studies have shed light on several ATN characteristics, such as scale-free structure^20^,^21^, “rich club” phenomena of airport hubs^25^,^26^, and existence of ‘free’ and ‘congested’ phases in flight traffic^27^. Motivated primarily from the observed multi-community structure of the global ATN^21^, we put forward the idea that, in order to understand the interactions between the ATNs of different regions, it is more useful to consider each region as a distinct subnetwork with inter-network connections among them — hence an *interacting network* model in lieu of a single network model. Critical structural differences in the ATNs^28^, and the fact that transatlantic flights mark a different regime (≈4000 km) in the distribution of geographical link lengths of the global ATN^29^ also lend further support to an interacting network picture in place of a single network.

The RAIN model statistically relates intra-network features to inter-network structure. Its fundamental framework is presented in a general fashion such that with appropriate formulation of its components, the larger framework is applicable to a wider spectrum of interacting real-world complex networks other than the particular example considered here.

## RAIN model

**Figure 1:**
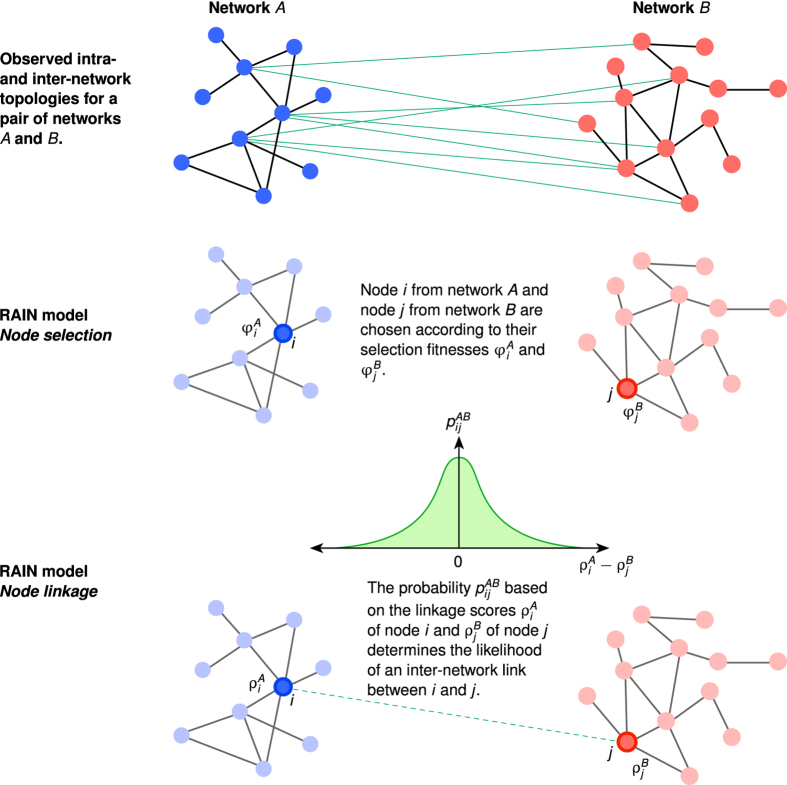
Schematic outline of the RAIN model showing two networks *A* and *B* as observed from the data (*Top row*), and how the RAIN model tries to model the inter-network links based on the observed intra-network features. The first *node selection* step (*Middle row*) involves the selection of two nodes *i* and *j*, one each from the given networks, based on their respective selection fitnesses *φ*_*i*_ and *φ*_*j*_. The second *node linkage* step involves the estimation of the linkage probability *p*_*ij*_ defined on the difference of the linkage scores *ρ*_*i*_ and *ρ*_*j*_ of nodes *i* and *j* (*Bottom row*). Based on this probability the RAIN model assigns a link (dashed green line) between nodes *i* and *j* in a single model run.

We assume that intra-network characteristics influence the interactions between different networks, and model this as follows: (i) a *selection step* which gives us a pair of nodes, one from each subnetwork, which might have an inter-network link, and (ii) a *linkage step* which determines whether the chosen nodes are connected. Both these steps are formulated in general terms and can be adapted to different situations. Node selection is based on the *selection fitness φ* of a node in its own network estimated from the data, which gives high (low) fitness to nodes of high (low) degree or any other node-based characteristic. Linkage is achieved by a *linkage probability p*, which may connect nodes with high (low) *linkage scores ρ* in one network to nodes with high (low) linkage scores in the other network, or vice versa, depending on the application. The linkage probability gives the probability that a pair of selected nodes are linked, given their linkage scores. A simple schematic outline of the steps involved in the RAIN model is given in Fig. 1 which shows how a pair of nodes are chosen and a link between them is assigned in a single model run. The particular form of selection fitness, linkage scores, and linkage probability used depends on the specific system being studied and the research questions being asked of it. Given the internal link distribution of each subnetwork, the RAIN model used with a suitable choice of fitness and linkage probabilities generates inter-network links and allows us to understand better the mechanisms underlying observed inter-network structure by using contesting, multiple scenarios.

### Node selection

Let **X**^*A*^ and **X**^*B*^ be the adjacency matrices of two networks *A* and *B* using which we can estimate a set of node-based characteristics 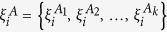 for a node *i* in network *A* and similarly another set of node-based characteristics 
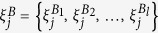
 for a node *j* in network *B*, where *k* ≠ *l* in general. The node-based measures 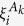 and 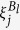 considered here could be typical measures such as degree, local clustering coefficient, local betweenness coefficient, page rank, eigenvector centrality, etc. Thus, the elements of the sets 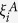 and 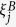 are *functions* defined on the nodes of the subnetworks. The indices *i* and *j* refer to nodes in networks *A* and *B* respectively from here on.

**Figure 2:**
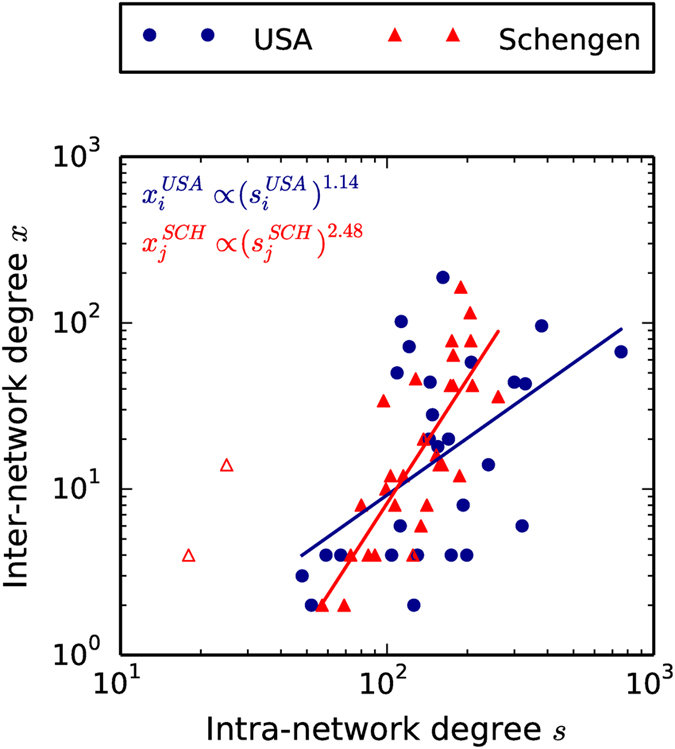
Intra- and inter-network degrees of the regional ATNs in USA (blue circles) and the Schengen (red triangles). The solid lines indicate the power law like relations estimated using linear regression between log(*x*) and log(*s*). Open markers denote outlying data points that are excluded from the regression analysis.

The RAIN model provides a statistical description of inter-network interactions as given by the inter-network adjacency matrix ***X***^*AB*^ based on the node-based characteristics 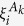 and 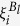. To do this, we first define a selection fitness *φ* for each node in network *A* such that,


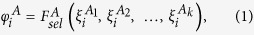


and similarly a selection fitness 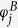 for each node in network *B*. We use *φ*_*i*_ and *φ*_*j*_ to preferentially select pairs of nodes from the given networks for a potential inter-network link. Nodes which are more “important” in a network receive a higher chance of having an inter-network link. The particular definition of importance based on 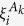 and 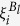 depends on beliefs/evidence about the factors that may influence inter-network interactions. For the example of the USA-Schengen airport networks, we choose to model the inter-network links as a power law function of the intra-network degrees *s*, based on an observed power law like dependence between the intra-network degrees *s* and the inter-network degrees *x* (cf. Fig. 2). Thus, we have


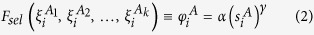


where the parameters *γ* and *α* are estimated from the data for each of the regional ATNs in the USA and the Schengen. We would like to caution here that the estimation of a statistical relation like the power-law should not be conflated with an explanatory mechanism between the intra- and inter-network degrees of the considered international airports. Clearly, the power-law is a simplified fit or a first approximation to the scatter of data points in Fig. 2. For the purpose of illustrating the functions of the RAIN model, however, this is helpful in showing how an overly simple choice of *F*_*sel*_ impacts the final results (see section Results below).

In other applications, prior understanding of the systems under study might motivate a different *F*_*sel*_, e.g. in case of the human brain, if we model each cortical region as an individual network, *φ* might depend not only on the intra-network degree but presumably also on other factors such as local clustering. The sets 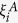 and 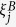 would then contain two elements (degree and clustering of nodes *i* and *j*) and not just the degree as in the current example. Moreover, *F*_*sel*_ might also likely have a different functional form, such as exponential, polynomial, or linear.

### Node linkage

The next step in the RAIN model is to determine whether or not to place an inter-network link between a pair of selected nodes based on a comparison of node-based features of the chosen nodes. For this, we define a *linkage score ρ* based on two sets of node-based characteristics 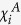 and 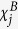,


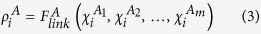


for a node *i* in network *A*, and a similar score 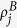 for a node *j* in network *B*. In Eq. 3, the set 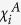 has *m* node-based measures whereas the set 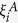 from Eq. 1 has *k* node-based measures. This highlights the fact that in general, the sets 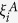 and 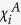 are not necessarily the same. However, in most cases we recommend having 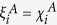 (and similarly 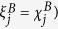 for a clearer interpretation of model results. In the case of the ATNs, 
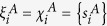
 and 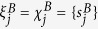, where *s*^*A*^ and *s*^*B*^ denote the intra-network degrees of nodes in networks *A* and *B* respectively. In particular, the linkage function *F*_*link*_ places the relative importances of nodes from both subnetworks on the same scale to make them comparable, for which we use a min-max transform where


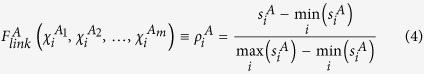


for a node *i* in network *A* and similarly we define 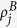 for a node *j* in network *B*. In general, one can use other functional forms of the linkage function such as a normalization to mean zero and unit standard deviation where the similarity of a pair of selected nodes (one from each network) is decided by how far (in units of standard deviation) they are situated from their respective means. The final ingredient of the RAIN model is a *linkage probability* 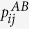 which operates on the linkage scores 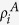 and 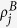 and returns the probability of placing a link between the selected nodes *i* and *j*, i.e.


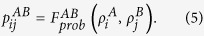


For the USA-Schengen ATNs, we choose a Gaussian probability such that airports with similar linkage scores (which, in turn, are proportional to the intra-degree *s* of the airport) have a higher chance of getting connected. This is motivated by the fact that hubs tend to be connected to other hubs in the global ATN. Thus,


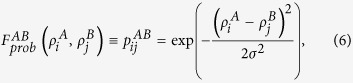


where *σ* is a free parameter of the model which influences the extent to which hubs tend to be connected to other hubs. Note that this choice of 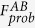 is assortative, but in general it is possible to define a disassortative linkage probability as well. For instance, in trying to model interactions between networks where the interaction topology is disassortative, we can potentially use 
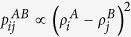
 which gives a higher chance to have inter-network links between those nodes *i* and *j* whose linkage scores are more different. Note, however, that the function 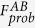 should be defined such that it maps into the interval [0, 1] and is well-defined on the entire range of linkage scores 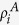 and 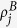.

## Inter-network assortativity

We consider two main characteristics of the interactions in order to assess the validity of the model outputs: (i) the inter-network degree distributions, and (ii) the inter-network assortativity between the linkage scores of nodes from both networks. We define the *inter-network assortativity coefficient r*^*inter*^ for two interacting networks as the Pearson correlation coefficient of linkage scores 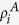 and 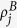 appearing at the opposite ends of inter-network links as given below:


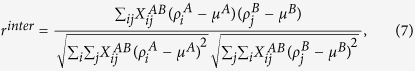


where 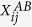 is (*i, j*)^*th*^ element of the inter-network adjacency matrix ***X***^*AB*^. In this matrix, the first index always corresponds to the network *A* and the second to the network *B*, and *μ*^*A*^ and *μ*^*B*^ are the mean values of *ρ* over inter-network links:


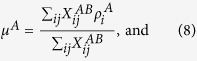



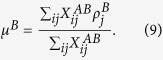


If on average the fitness values at the two ends of inter-network edges are similar, then *r*^*inter*^ > 0, if they tend to vary in opposite directions then it is negative and if no such correlation exists, it is zero. This measure, ranging between −1 and 1, reflects the tendency for high (low) degree nodes from one subnetwork to be connected to high (low) degree nodes in the other subnetwork, or vice versa. Note that the inter-network assortativity defined here is similar to the inter degree-degree correlation (IDDC) defined in an earlier study^30^. However, a major difference between the two is that the IDDC is defined specifically on the degrees of the nodes, whereas the inter-network assortativity can be used to estimate the assortative nature of any node-based characteristic. In our current example, we estimate *r*^*inter*^ for the linkage scores 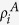 and 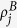 which are, in effect, normalized degrees.

## Results

### ATN construction

We use flight route data of the global ATN from www.openflights.org and determine the flight routes within USA and within the 26 Schengen countries, as well as the flights between the USA and the Schengen area. A total of 2,991 airports are obtained of which 1,643 are in the USA and 1,348 are in the Schengen region. The regional ATNs have a total of 10,673 flights in the USA and 9,714 flights in the Schengen area. Compared to this, there are only 919 transatlantic flights between 28 international airports in the USA and 31 in the Schengen. Although flight routes are inherently directed, we represent them as undirected edges in our analysis. Multiple flights between two airports are retained as multiple edges. The total number of regional (transatlantic) flights starting or ending at a given airport gives us the intra-network (inter-network) degree.

### Intra- and inter-network degrees

The inter-network degree *x* of an airport follows a power law like relation based on its intra-network degree *s* (Fig. 2). We note that this is only a first approximation of the various factors that could possibly influence the inter-network degree. This is particularly true for the case of the USA ATN, where the power law curve does not fully explain the relation between *x* and *s*. This discrepancy between the fitted curve and the unknown true relation between the intra- and inter-network degrees has further consequences in the results of the RAIN model (discussed in the following sections). We estimate the power law relation as stated in Eq. 2 using linear regression between log(*x*) and log(*s*), and find that *γ* = 1.14 for USA but *γ* = 2.48 for the Schengen. We use this to define the selection fitnesses *φ*^*USA*^ and *φ*^*SCH*^ for all airports in both networks such that 
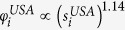
 and 
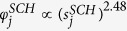
. The scatter plot shown in Fig. 2 includes the intra- and inter-network degrees of only those nodes which have at least one transatlantic link, which results in 28 airports from the USA and 31 airports from the Schengen. Two airports from the Schengen region with *s* < 50 are considered as outlying points and are excluded from the regression analysis because they have much higher inter-network degrees while only relatively low intra-network degrees.

### RAIN model for USA-Schengen interactions

To implement the RAIN model for USA-Schengen inter-network interactions, we consider three different scenarios:

- We select nodes from each network based on 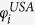 and 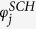, but we do not consider linkage scores for link placement. Instead, we put a link between all selected nodes. We call this *only preferential picking*, i.e. *onlyPP*.
- We select transatlantic nodes with a uniform probability disregarding their selection fitness values, but place a link only when allowed by the probability *p*_*ij*_ based on their linkage scores 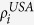 and 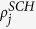. We call this *only assortative matching*, i.e. *onlyAM*.
- We select nodes based on selection fitness values and place a link based on linkage scores and the linkage probability. We call this the *full model*, i.e. *PPAM*.

**Figure 3:**
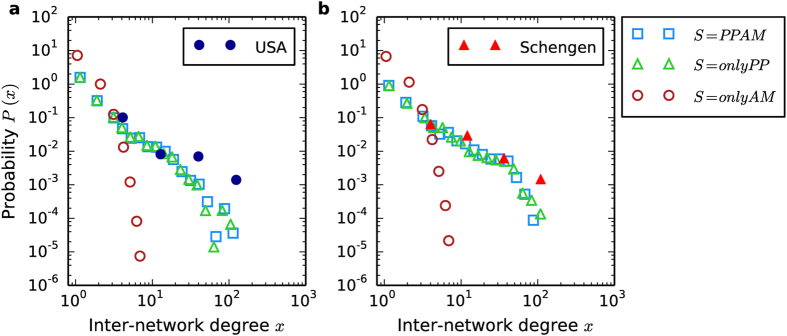
Inter-network degree distributions from the RAIN model (open markers) and the data (filled markers) for (**a**) USA and (**b**) Schengen regions. The three model scenarios are shown for both regions — *S* = *PPAM* (open blue squares), *S* = *onlyPP* (open green triangles) and *S* = *onlyAM* (open red circles). The model degree distributions for the *onlyPP* and the *PPAM* scenarios are almost identical and relatively well approximate the power law like distributions form the data except the tail portions. On the other hand, the *onlyAM* scenario fails to reproduce the observed distributions entirely. The width of the linkage probability is 0.8 for the above plots. We construct these distributions with logarithmic binning of *x* where the number of bins is given by Sturges’ Formula.

We use *S* = {*onlyPP, onlyAM, PPAM*} to denote the set of the three possible scenarios considered in our analysis. In each model run, we generate 919 transatlantic links which is equal to the observed number in the data. Moreover, we consider 5000 such model runs for each scenario (cf. Methods). In Fig. 3, we show the inter-network degree distributions generated by the RAIN model for the various scenarios. We mainly find that *S* = *onlyAM* fails to replicate the observed inter-network degree distributions, whereas the *onlyPP* and *PPA* scenarios have almost overlapping distributions — both of which somewhat approximate the observed distributions from the data, barring the tail regions. The distributions match better for the Schengen region than that for the USA, indicating that the *s*^*SCH*^ has a better explanatory power for the inter-network degree *x*^*SCH*^ in the Schengen and that there are additional factors influencing the inter-network degree for airports in USA other than *s*^*USA*^.

**Figure 4:**
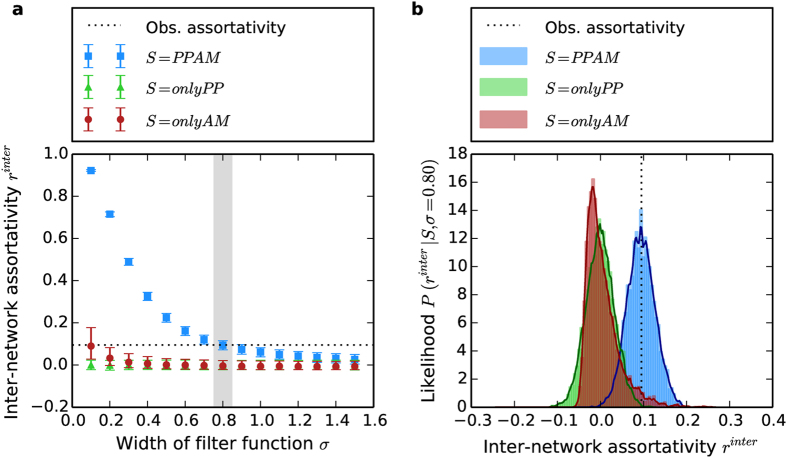
Inter-network assortativity *r*^*inter*^ from the RAIN model for different values of *σ* (**a**) and the likelihood of obtaining the observed inter-network assortativity *P*(*r*^*inter*^|*S, σ* = 0.8) for the different model scenarios at *σ* = 0.8 (**b**). The error bars in **a** denote the median *r*^*inter*^ along with the IQR for the three model scenarios: (i) *S* = *onlyPP* (green circles), (ii) *S* = *onlyAM* (red triangles), and (iii) *S* = *PPAM* (blue squares). The probability densities in **b** are obtained using a normalized histogram of 5000 model runs (shaded regions), along with a kernel density estimation (solid lines) using an optimal bandwidth for exponential kernels with the Python toolkit Scikit-learn^31^. The dotted lines in both subplots denote the observed inter-network assortativity value 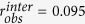. The grey shaded area in **a** highlights the value of *σ* = 0.8 which was used to estimate the results in **b**.

**Figure 5:**
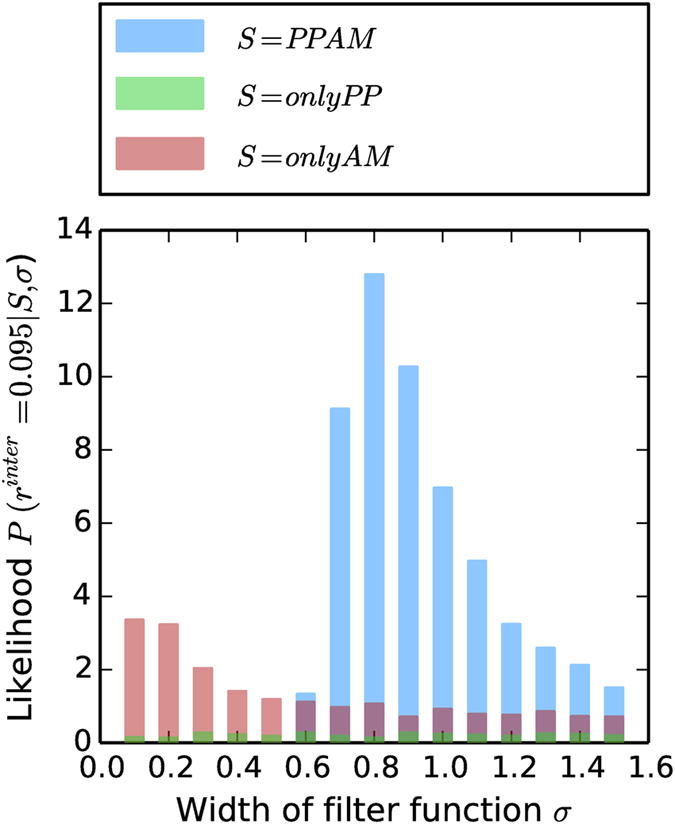
Likelihood of obtaining 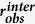 given a model scenario for different values of σ. The height of the different colored bars at each value of *σ* is equal to the height of the estimated probability density *P*(*r*^*inter*^|*S, σ*) at 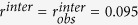. The likelihood *P*(*r*^*inter*^ = 0.095|*S, σ*) at each *σ* value is estimated as shown in Fig. 4b.

We estimate the median *r*^*inter*^ values along with its inter-quartile range (IQR) from 5000 model runs for various values of *σ* (Fig. 4a). There are two cases for which the median *r*^*inter*^ value from the RAIN model scenarios are close to the observed 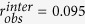: (a) *σ* = 0.1 and *S* = *onlyAM*, and (b) *σ* = 0.8 and *S* = *PPAM*. From the *r*^*inter*^ values obtained for each scenario, we obtain the distributions of *r*^*inter*^ (Fig. 4b), which we interpret as the likelihood *P*(*r*^*inter*^|*S, σ* = 0.8) of obtaining a chosen inter-network assortativity value given a particular model scenario at *σ* = 0.8. We estimate similarly the likelihood of getting 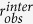 for each scenario given *σ*, i.e. *P*(*r*^*inter*^|*S, σ*) when 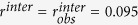, which is shown in Fig. 5. We note several points from Fig. 5: (a) the *PPAM* scenario has the maximum likelihood over all values of *σ* and over all model scenarios at *σ* = 0.8, (b) the *PPAM* scenario shows a wide range of *σ* values for which it has the maximum likelihood as compared to the other scenarios, and (b) even though Fig. 4a indicated the *onlyAM* scenario at *σ* = 0.1 as a potential candidate that gives a value of *r*^*inter*^ close to the observed value, the likelihood of the *onlyAM* scenario at that value *σ* is nearly three times as less than the likelihood of getting the observed assortativity with the *PPAM* scenario at *σ* = 0.8.

## Discussion

In this study, we have proposed a random interacting network (RAIN) model and used it to model the transatlantic interactions between the USA and Schengen flight networks. A crucial feature of the model is that it enables us to relate *intra*-network characteristics to *inter*-network structure (as seen in Eqs. 1 and 3). Being a model of network interactions, it inherently allows us to define regional (intra-network) hubs and formulate measureable, well-defined inter-network characteristics such as the inter-network degree distributions and inter-network assortativity. The RAIN model is essentially a prescription to obtain inter-network links between any given pair of individual networks such that they are determined by intra-network features. It requires two consecutive steps: node selection and node linkage. *Node selection* is defined using the selection fitness *φ* of a node, based on any number of node-based characteristics such as degree, page rank, local betweenness, and local clustering. The appropriate set of node-based characteristics required to explain the inter-network structure could be, in principle, decided *a priori* based on observations of the complex networks under study. *Node linkage* in the RAIN model is achieved with the help of linkage scores *ρ* defined for each node on the basis of a function *F*_*link*_. The primary purpose of *F*_*link*_ is to transform the relative importances of nodes form both subnetworks to a similar scale such that they become comparable. The precise notion of what constitues the ‘importance’ of a node has to be decided *a priori*. In most cases, this is equal to the node-based characteristics that determine *φ*. However *F*_*sel*_ could have a different functional form than *F*_*link*_ as is the case of our chosen example with the USA-Schengen ATNs. Also necessary for the linkage between any pair of nodes is the linkage probability *p*_*ij*_ which returns a probability of how likely it is that selected nodes *i* and *j* with linkage scores *ρ*_*i*_ and *ρ*_*j*_ are linked. Different choices of *p*_*ij*_ could lead to different inter-network topologies.

In applying the RAIN model to the USA-Schengen ATN interactions, we have started from the fundamental assumption that the number of transatlantic flights of any given airport (i.e. the inter-network degree *x*) is partly determined by the number of flights that the airport has in its own regional network (i.e. the intra-network degree *s*). Estimating the selection fitness for each airport from the data, we have shown that the inter-network degree of an airport, on average, depends on its intra-network degree in a power law like fashion, with exponents of 1.14 and 2.48 for airports in USA and Schengen respectively. However, this relation between *x* and *s* is only a rough approximation of the various factors that might influence the inter-network degree of an airport. This is seen from the fact that the selection fitness used in our study failed to reproduce the tail portions of the inter-network degree distributions as obtained from the data, especially for USA (Fig. 3). In general, *x* can depend on any combination of various node-based characteristics such as geographical location, proximity to other airports, intra-network betweenness, intra-network clustering, etc. A more thorough application of the RAIN model might aim to unravel the correct combination of such influencing factors on *x* and define a meaningful *F*_*sel*_ (as in Eq. 1) based on a larger set of 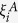
’s and 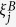
’s. Such an analysis, however, is beyond the scope of our current study.

We have also considered the inter-network assortativity *r*^*inter*^ as an additional critical characteristic of the interaction topology. In the model implementation, we have chosen three model scenarios: *S* = {*onlyPP, onlyAM, PPAM*} and have shown that the likelihood of obtaining the observed inter- network assortativity 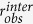 was the largest for *S* = *PPAM*, which included both preferential node selection and assortative node linkage (Fig. 5). Even though *S* = *onlyPP* was able to reproduce the inter-network degree distributions as well as *S* = *PPAM*, the latter had much higher likelihoods of producing assortativity values close to 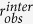. On the other hand, even though *S* = *onlyAM* was able to produce assortativity values close to 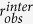 for low values of *σ* at around 0.1, it failed to reproduce the inter-network degree distributions of the real-world data (cf. Fig. 3). According to the principle of Occam’s razor, the *miminal* model with the power to relatively well explain both the inter-network degree distributions *as well as* the inter-network assortativity is the full model scenario *PPAM*. Thus, the existence of a transatlantic flight between a pair of airports, chosen one each from USA and Schengen, depends on two major factors, (a) the number of regional flights to- and-from the airports, and (b) the similarity between the relative importance of the airports in their respective regional ATNs. Both these factors are necessary to reproduce the observed interacting network characteristics.

The RAIN model can incorporate arbitrarily complex inter-network structures and with appropriate choices of the selection fitness, linkage scores, and linkage (filter) function, it has the potential to be applied to a wide variety of different real-world systems that are modeled as complex networks. Other systems where this framework is applicable include the neuronal network of the brain, protein interaction networks, and power grid networks. However, there is no universal prescription to define *F*_*sel*_, *F*_*link*_, and *F*_*prob*_, as well as to decide which intra-network characteristics should be chosen to model the inter-network topology. These details have to be worked out and estimated with respect to each individual analysis. The application of this framework to other complex systems with the hope of uncovering newer insights is the focus of future studies.

## Methods

### RAIN model implementation for the USA-Schengen ATN example

#### Node selection based on preferential picking

We implement node selection, or preferential picking (PP), using a standard method of generating network links on the basis of fitness scores that is free from rejections. Given that the selection fitness scores 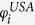 and 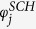 of all nodes *i* in USA and *j* in the Schengen have been estimated according to Eq. 2, the algorithm for PP implemented in our analysis is outlined below.

PP 1. Define 
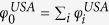
 and 
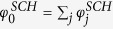
.

PP 2.Construct the interval 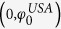 from *N* smaller, non-overlapping, continuous intervals as,


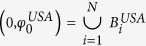


where


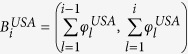


such that the component intervals 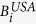 are placed end-to-end and the length of the *i*^*th*^ component interval denotes the fitness of node *i*, i.e., 
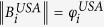
. Here *N* = 1643 is the total number of airports in the USA. Repeat this similarly for the Schengen region with *N* = 1348 and using 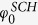.

PP 3. Generate random numbers *r*^*USA*^ in the interval 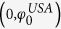 and *r*^*SCH*^ in the interval 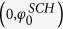.

PP 4. Identify the component intervals 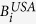 and 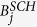 in which *r*^*USA*^ and *r*^*SCH*^ lie.

PP 5. Select nodes *i* from USA and *j* from the Schengen for a potential transatlantic link.

#### Node linkage based on assortative matching

We implement the node linkage based on assortative matching (AM) based on the Gaussian linkage probability *p*_*ij*_ given a particular linkage probability width *σ* as follows.

AM 1. Generate a random number *r* in the interval (0, 1).

AM 2. Evaluate the Gaussian linkage probability *p*_*ij*_ based on the linkage scores 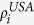 and 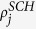 of nodes *i* and *j* according to Eq. 4.

AM 3. If *r* < *p*_*ij*_ then place a link between nodes *i* and *j*, else do nothing (i.e., reject the choice of *i* and *j* for a transatlantic edge).

#### Model scenarios

In our analysis we consider, three model scenarios: *S* = {*onlyPP, onlyAM, PPAM*}. The details of the algorithmic implementation of these scenarios for a single model run is outlined below.

- *S* = *onlyPP.* First, choose nodes *i* from USA and *j* from the Schengen using the steps PP 1–5 outlined above. Next, place a transatlantic link between *i* and *j* irrespective of their linkage scores 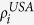 and 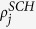.
- *S* = *onlyAM.* First choose nodes *i* from USA and *j* from the Schengen randomly, i.e., irrespective of their selection fitnesses 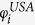 and 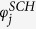. Next, place a transatlantic link between *i* and *j* according to their linkage scores 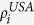 and 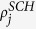 and the linkage probability *p*_*ij*_ using steps AM 1–3 outlined above.
- *S* = *PPAM.* First, choose nodes *i* from USA and *j* from the Schengen using the steps PP 1–5 outlined above. Next, place a transatlantic link between *i* and *j* according to their linkage scores 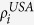 and 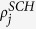 and the linkage probability *p*_*ij*_ using steps AM 1–3 outlined above.

For each of the model scenarios given above, the above steps are repeated until the number of transatlantic edges in the model are equal to 919, which is the number of transatlantic flights observed in the data.

### Likelihood of obtaining 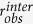


We run the RAIN model for each of the three scenarios for 5000 runs for *σ* varying from 0.1 to 1.5 in steps of 0.1. For each model scenario, given a particular value of *σ*, we pool together all the degree sequences to get an overall degree distribution from the RAIN model for that scenario. This is shown in Fig. 3 for *σ* = 0.8. Furthermore, we estimate *r*^*inter*^ for each model run, for each scenario, and for each *σ*. This is used to estimate the probability density *P*(*r*^*inter*^|*S, σ*) (cf. Fig. 4) based on a kernel density estimation using an optimal bandwidth for exponential kernels with the Python toolkit Scikit-learn^31^. The likelihood can also be used to estimate the most likely model scenario given that we observe 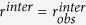 in the real-world data,


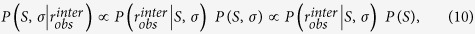


where *P*(*S*) is our prior belief on how likely it is to have the chosen scenario (from the set *S*) in the first place. Note that, taking into account the fact that the only AM scenario fails to reproduce the inter- network degree distributions, we could set the priors for each scenario such that 0 < *P*(*S* = *onlyAM*) ≪ *P*(*S* = *onlyPP*) < *P*(*S* = *PPAM*) < 1, based on which we can determine the most likely model given the data. For such choices of priors, *S* = *PPAM* is the most likely model given the data.

## Additional Information

**How to cite this article**: Goswami, B. *et al*. A random interacting network model for complex networks. *Sci. Rep.* **5**, 18183; doi: 10.1038/srep18183 (2015).
